# ACAT1 regulates tertiary lymphoid structures: A target for enhancing immunotherapy in non–small cell lung cancer

**DOI:** 10.1172/JCI191094

**Published:** 2025-04-01

**Authors:** Sophie O’Keefe, Qiwei Wang

**Affiliations:** 1 Department of Microbiology, Immunology, and Cancer Biology, University of Virginia School of Medicine, Charlottesville, Virginia, USA.; 2 University of Virginia Comprehensive Cancer Center, Charlottesville, Virginia, USA.; 3 Beirne B. Carter Center for Immunology Research, University of Virginia School of Medicine, Charlottesville, Virginia, USA.

## Abstract

Non–small cell lung cancer (NSCLC), the most common type of lung cancer, remains a leading cause of cancer-related mortality worldwide. Immune checkpoint inhibitors (ICIs) have emerged as a promising therapy for NSCLC but only benefit a subset of patients. In this issue of the *JCI*, Jiao et al. revealed that acetyl-CoA acetyltransferase 1 (ACAT1) limited the efficacy of ICIs in NSCLC by impeding tertiary lymphoid structures (TLS) in the tumor microenvironment (TME). Targeting ACAT1 in tumor cells reduced mitochondrial hypersuccinylation and oxidative stress, enhancing TLS abundance and improving the efficacy of ICIs in preclinical murine models of NSCLC.

## Harnessing TLS to enhance immunotherapy efficacy in NSCLC

**Figure 1:**
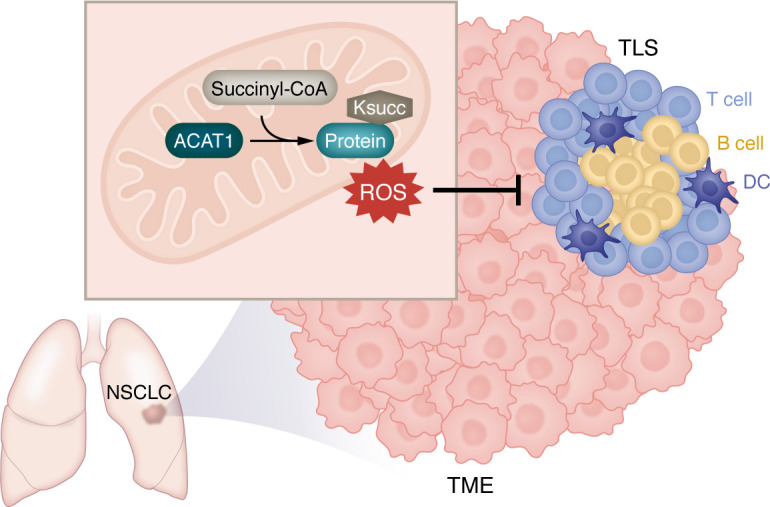
Acetyl-CoA acetyltransferase 1 functions as a metabolic regulator of tertiary lymphoid structures in non–small cell lung cancer. Tumor cell–intrinsic Acetyl-CoA acetyltransferase 1 (ACAT1) interacts with succinyl-CoA, driving hypersuccinylation at lysines (Ksucc) of mitochondrial proteins, which enhances intratumoral reactive oxygen species (ROS). This oxidative stress suppresses B cells, thereby inhibiting the formation of tertiary lymphoid structures (TLS) in the tumor microenvironment (TME). NSCLC, non–small cell lung cancer; DC, dendritic cell.

Tertiary Lymphoid Structures (TLS) are organized aggregates of immune cells that develop in nonlymphoid tissues (1). In patients with non–small cell lung cancer (NSCLC), tumor-associated TLS contain clusters of dendritic cells (DCs), T cells, and B cells (2, 3). Previous studies have shown that a high density of follicular B cells (2) and mature antigen-presenting DCs (3) in tumor-associated TLS is correlated with long-term survival of patients with NSCLC. Moreover, Vanhersecke et al. report that the presence of mature TLS (defined by the presence of CD23-positive follicular DCs) predicts an improved response to ICIs in multiple cancers, including NSCLC, independent of PD-L1 expression (4). These findings highlight the potential of modulating TLS to enhance immunotherapy efficacy in NSCLC. Notably, strategies to harness TLS are still evolving (5). Using preclinical murine models of lung adenocarcinoma (LUAD), Ng et al. show that LUAD-associated TLS can be induced by an intranasal plasmid encoding *Cxcl13* or a KRAS^G12C^ inhibitor, MRTX-849 (6). These discoveries suggest the feasibility of and potential strategies for exploiting TLS in lung cancer. In this issue of the *JCI*, Jiao et al. studied how cancer metabolism regulates TLS in NSCLC (7) (Figure 1). Metabolic reprogramming is a hallmark of cancer (8), and identifying metabolic regulators of TLS could provide targets for developing therapeutic strategies to harness TLS in a broader patient population.

## ACAT1 acts as a metabolic regulator of TLS

Acetyl-CoA acetyltransferase 1 (ACAT1) is a mitochondrially localized enzyme that plays a critical role in mitochondrial metabolism by regulating protein functions mainly via acetylation, a major type of posttranslational modification (PTM) that occurs in more than 40% of mitochondrial proteins (9). Previous studies have revealed the role of ACAT1 in controlling tumor growth. For instance, Fan et al. report that oncogenic tyrosine kinases induce the tetramerization and activation of ACAT1, which facilitates the acetylation of pyruvate dehydrogenase (PDH) and PDH phosphatase, promoting glycolysis in cancer cells and fueling tumor growth (10). More recently, two studies from Youjun Li’s group suggest that ACAT1-mediated acetylation promotes tumor growth by regulating tumor cell lipid metabolism in preclinical models of colorectal cancer and hepatocellular carcinoma (11, 12). These studies highlight the potential of ACAT1 as a therapeutic target for cancer treatment, emphasizing its cell-autonomous role in tumor growth. However, the role of ACAT1 in the tumor microenvironment (TME) is unclear.

Here, Jiao et al. demonstrated that tumor cell–intrinsic ACAT1 in NSCLC conferred resistance to ICI therapy by impeding TLS formation (7). Using Lewis lung carcinoma (LLC), a syngeneic murine model of lung cancer, Jiao et al. conducted a metabolism-focused in vivo CRISPR screen coupled with ICI treatment to identify tumor cell–intrinsic metabolic regulators that affect the antitumor efficacy of ICIs. Notably, ACAT1 was among the top ten genes whose depletion improved the response to ICI therapy. Interestingly, ACAT1 was also highly expressed in patients with NSCLC who had a low TLS signature. These data suggest that targeting ACAT1 may enhance ICI efficacy and promote TLS.

Jiao et al. then validated these findings in both *Kras^G12D^/Trp53^−/−^* (KP) and LLC orthotopic lung tumor models deficient in *Acat1* (7*)*. They demonstrated that knocking down *Acat1* promoted TLS formation in the TME and enhanced the efficacy of ICI. Importantly, inducible knockdown of tumor cell *Acat1* in established KP tumors showed similar results, suggesting the therapeutic potential of targeting *Acat1*.

To investigate how *Acat1* knockdown improved TLS formation, the authors performed single-cell RNA-seq (scRNA-seq) and flow cytometry and determined that *Acat1* knockdown led to increased B cell clusters and more CD4^+^ T follicular helper (TFH) cells in the TME. Importantly, depleting B cells with an anti-CD20 antibody or inhibiting TLS formation with TAK-799 (also known as TAK-779), an inhibitor of CCR5 and CXCL13/CXCR5, abolished *Acat1* knockdown–induced TLS formation and tumor control. Moreover, analysis of tumor samples from patients with lung cancer as well as TCGA datasets confirmed that TLS increased in NSCLC tissues with lower ACAT1 and correlated with better immunotherapy outcomes. Collectively, Jiao et al. demonstrated the role of tumor cell–intrinsic ACAT1 as a metabolic target to improve TLS formation, thereby enhancing the efficacy of ICI therapy (7).

Thus, Jiao et al. revealed another role of ACAT1 in modulating TLS (7). Combined with its well-established cell-autonomous role in driving tumor cell proliferation (10), the enzyme ACAT1 could fuel dual mechanisms to promote cancer.

## ACAT1-mediated hypersuccinylation inhibits TLS formation through oxidative stress

To further investigate the mechanisms by which ACAT1 influences TLS formation, Jiao, Guo, and colleagues analyzed ACAT1-binding proteins by conducting ACAT1 immunoprecipitation and mass spectrometry (7). Notably, they discovered that ACAT1-binding proteins in carcinoma tissues from patients with lung cancer were hypersuccinylated rather than acetylated. Moreover, these ACAT1-interacting, hypersuccinylated proteins were mainly clustered in mitochondria metabolic pathways (7). Indeed, protein acetylation and succinylation are closely related processes, both of which can be catalyzed by lysine acetyltransferases, such as lysine acetyltransferase 2A (KAT2A) (13). However, it is unclear whether ACAT1 can catalyze lysine succinylation. Jiao et al. demonstrated that ACAT1 interacted with succinyl-CoA (SCoA) and acted as a mitochondrial lysine succinyltransferase, mediating mitochondrial protein succinylation (7).

Succinylation is an important type of PTM, characterized by transfer of a negatively charged succinyl group (-CO-CH_2_-CH_2_-CO_2_H) to lysine residues of the targeted proteins (14). Succinylation of proteins might occur enzymatically by succinyltransferases, such as KAT2A and carnitine palmitoyltransferase 1A (CPT1A), or nonenzymatically via succinyl-CoA formed in the tricarboxylic acid cycle (13, 14). Lysine succinylation has important cellular functions. Recent studies have shown that lysine succinylation of mitochondrial proteins, such as serine β-lactamase–like protein (LACTB), enhances mitochondrial functions and cancer cell proliferation, ultimately facilitating hepatocellular carcinoma progression (15). However, the effects of lysine succinylation on tumor immunology and cancer immunotherapy remains elusive. Jiao et al. demonstrated that ACAT1-mediated mitochondrial hypersuccinylation (such as that of mitochondrial trifunctional enzyme subunit α, HADHA), but not acetylation, promoted tumor cell mitochondria oxidative metabolism (7). Notably, the authors found that ACAT1-dependent tumor cell–derived reactive oxygen species (ROS) limited B cell viability and reduced TLS formation. In both KP and LLC preclinical murine models of lung cancer, treatment with the ROS scavenger NAC overcame ACAT1-mediated inhibition of TLS and suppressed tumor growth. Thus, Jiao et al. demonstrated that tumor cell ACAT1 suppressed B cell viability and reduced TLS formation by elevating intratumoral oxidative stress, which was driven by ACAT1-induced mitochondrial hypersuccinylation in tumor cells (7).

## Unanswered questions and future directions

Jiao et al. presented compelling evidence identifying ACAT1 as a therapeutic target to harness TLS and improve the efficacy of immunotherapy in NSCLC (7). Their data convincingly showed that the loss of ACAT1 in tumor cells promoted B cell viability and TLS formation. However, the mechanisms by which ACAT1 inhibition affects the composition and function of immune cells within the TLS, and how it improves ICI efficacy, remain incompletely understood, as these effects may not be fully explained by the reduced oxidative stress alone. Moreover, Jiao et al. have notably conducted a thorough investigation into tumor cell–intrinsic ACAT1 (7). To enhance translational potential, the role of ACAT1 in noncancerous host cells, such as immune cells and cancer-associated fibroblasts (CAFs), needs further evaluation. For example, Jiao et al. showed that ACAT1-proficient LLC cells grow faster in ACAT1^–/–^ mice compared with ACAT1^+/+^ mice, indicating the complexity of pharmacologically targeting ACAT1 (7). Investigating how ACAT1-mediated PTMs and metabolic pathways influence the functions of host noncancerous cells could aid in designing rational combination therapies to harness the antitumor immunity of TLS. Finally, future studies should evaluate whether pharmacologically targeting ACAT1 can leverage the antitumor immunity of TLS in NSCLC. Beyond combining ACAT1 inhibition with ICIs, it would also be beneficial to explore its combination with agents that may enhance the formation and antitumor activity of TLS, such as KRAS^G12C^ inhibitors (6), STING agonists (16), or LIGHT/TNFSF14-based therapies (17).

## Version 1 04/01/2025

Electronic publication
